# Lateral Sagittal Versus Costoclavicular Approaches for Ultrasound-Guided Infraclavicular Brachial Plexus Block: A Comparison of Block Dynamics Through A Randomized Clinical Trial

**DOI:** 10.7759/cureus.14129

**Published:** 2021-03-26

**Authors:** Burhan Dost, Cengiz Kaya, Yasemin B Ustun, Esra Turunc, Sibel Baris

**Affiliations:** 1 Anesthesiology and Reanimation, Ondokuz Mayis University, Samsun, TUR

**Keywords:** brachial plexus, nerve block, ultrasonography, upper extremity, patient satisfaction

## Abstract

Introduction

In this study, our objective was to compare the lateral sagittal infraclavicular block (LS-ICB) with the costoclavicular infraclavicular block (CC-ICB) for ultrasound (US)-guided infraclavicular brachial plexus block in terms of block dynamics as well as patient and surgeon satisfaction levels.

Methods

A total of 100 patients, falling under the American Society of Anaesthesiologists (ASA) I-III categories, who were aged 18-65 years and scheduled for elective forearm and hand surgery were enrolled in the study. The patients were randomly allocated to receive a US-guided LS-ICB or US‑guided CC-ICB. The local anesthetic (LA) agent used (20-ml 0.5% bupivacaine) was identical in all subjects. The block performance time and the motor and sensory block onset times were determined to be the primary outcomes.

Results

The block performance time and the sensory block onset time were shorter in the CC-ICB group compared to the LS-ICB group [median (interquartile range): three (2.5-3.3) vs. two (1.5-2.3) minutes, p: <0.001; five (4.4-6) vs. four (3.8-6) minutes, p = 0.022, respectively]. The number of needle redirections was lower in the CC-ICB [three (2.7-4) vs. two (one to two) times, p: <0.001]. The motor block onset time and the motor-sensory block times were similar in both groups. There were more patients with a complete sensory blockade at five and 10 minutes in the CC-ICB group than in the LS-ICB group (30% vs. 12%, p = 0.027; 66% vs. 26%, p: <0.001, respectively). No complications were observed with regard to both techniques, and patient and surgeon satisfaction levels observed were similar for both groups.

Conclusion

Based on our findings, the CC approach provided a shorter performance time and a faster onset of the sensory block compared to the LS approach. However, no complications were reported with respect to either technique, and similar patient and surgeon satisfaction levels were observed.

## Introduction

The ultrasound (US)-guided infraclavicular block (ICB) is commonly used to provide anesthesia and analgesia in the arm, elbow, forearm, and hand surgeries. The ICB blocks the brachial plexus at the level of the cords [1,2], and a lateral sagittal (LS) approach is usually preferred for this block. In the LS approach, the block needle is placed in the lateral infraclavicular fossa in the parasagittal plane, and a local anesthetic (LA) is injected around the second part of the axillary artery. Despite being a safe and effective technique, the LS approach may require multiple injections and the use of relatively large volumes of LA, since the cords are located deeply and separately from each other and have many anatomical variations [1,3,4].

By contrast, the costoclavicular (CC) area is located posterior to the clavicle in the proximal infraclavicular fossa and is situated between the subclavius anteriorly and the serratus anterior muscle in the posterior. In this area, the cords are always located lateral to the axillary vessels and very close to each other. Therefore, the more superficial location of the CC compared to the LS approach makes the CC area more practical for ICB [5]. Karmakar et al. have recently described a CC approach using a single injection and a relatively small volume of anesthetic and shown that the CC area may be more advantageous [6]. However, two later studies obtained conflicting results regarding the sensory and motor block onset times when LS-ICB and CC-ICB were compared [7,8]. This variance was attributed to the difference in the LA used and its volume. In light of these facts, the aim of the present study was to compare the block dynamics related to LS-ICB and CC-ICB as well as patient and surgeon satisfaction levels more comprehensively by using a lower volume of LA.

## Materials and methods

Study design

This was a prospective, randomized, double-blind study conducted between May and October 2020 after obtaining ethics committee approval from the Ondokuz Mayis University Clinical Research Ethics Committee (approval no: 2020/36). The study was registered on clinicaltrials.gov prior to patient enrollment (NCT04356521).

Study population

**Figure 1:**
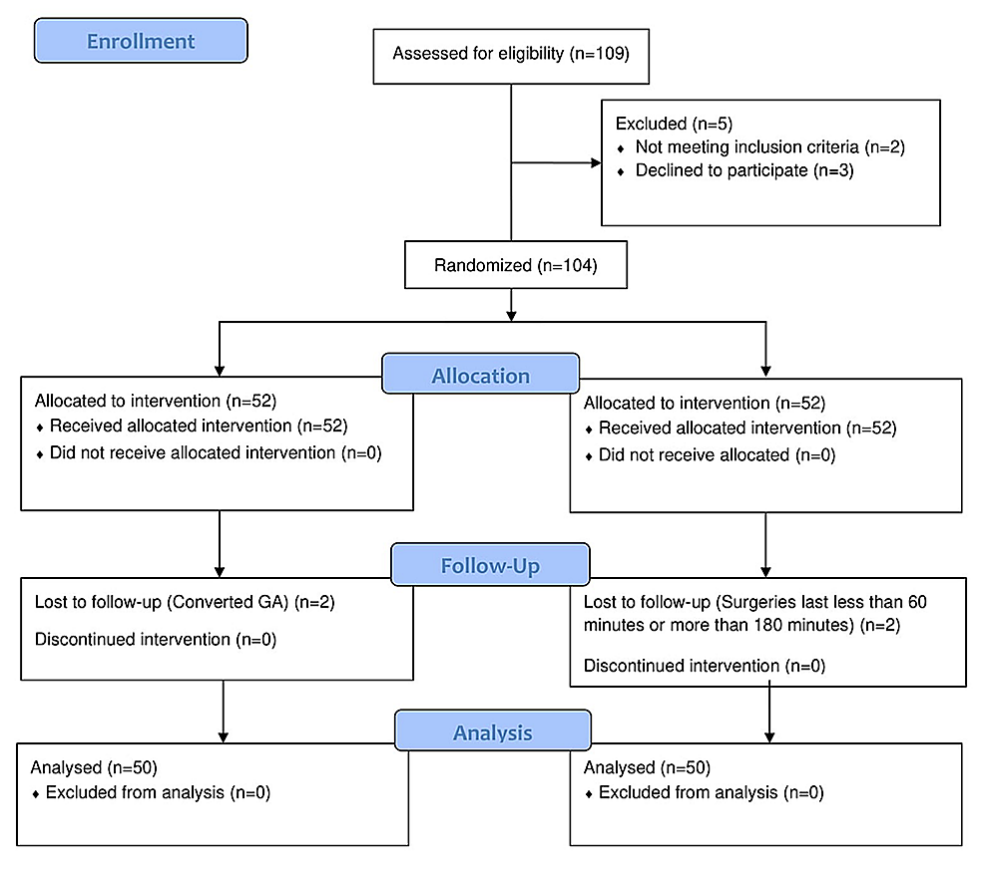
Flow diagram of patient data distribution GA: general anesthesia

Of the 109 patients screened for recruitment, 100 adult patients aged 18-65 years who were scheduled for forearm and hand surgery with the American Society of Anaesthesiologists (ASA) grades I-III and gave written informed consent were enrolled in this study (Figure 1).

The exclusion criteria were as follows: obesity (BMI of >30 kg/m^2^); regional anesthesia contraindications (patient's refusal to participate and infection over the infraclavicular fossa); severe renal, cardiac, or hepatic disease; a history of hypersensitivity or allergy to LA; opioid or steroid use for more than four weeks; psychiatric disorders; undergoing analgesic therapy within the last 48 hours preoperatively; operations lasting less than 60 minutes or more than 180 minutes; and patients who converted to general anesthesia.

Randomization

Patients were randomized using a computer-generated randomization ID. The ID created for each patient was conveyed in a sealed envelope by an independent assistant to the anesthesiologist who was to perform the ICB. The blocks were performed by experienced anesthesiologists who had previously performed at least 50 successful blocks without complications. The outcome assessor (anesthesia resident) who performed the sensory-motor assessment after the ICB was not present in the regional anesthesia room during the block placement and was blind to group allocation. The block performance time, number of needle redirections, number of needle attempts, and number of patients who required a rescue block were not blinded.

Preoperative preparation

The patients were premedicated with 0.03 mg/kg midazolam and monitored with standard ASA monitoring procedures (electrocardiography, noninvasive arterial pressure, and peripheral oxygen saturation). All patients received 4 L/minute nasal oxygen in the regional anesthesia room. Remifentanil infusion was administered at 0.05-0.1 mcg/kg/minute to maintain a Ramsay Sedation Scale (RSS) score of 2 (awake, calm, watching the surroundings) during the block and throughout the surgery.

Block performances

A high-frequency linear US (Logiq V1, 8-13 MHz, GE Healthcare, Chicago, IL) probe was adjusted to a depth of 4-6 cm. A 21G short bevel 80-mm needle (Stimuplex® Ultra 360®, B. Braun, Germany) was chosen as the needle. Both blocks were performed with the patients in the supine position and under aseptic conditions; 0.5% bupivacaine was used as the LA without any additives. The in-plane technique was used for both blocks.

Lateral sagittal approach (Group LS)

**Figure 2:**
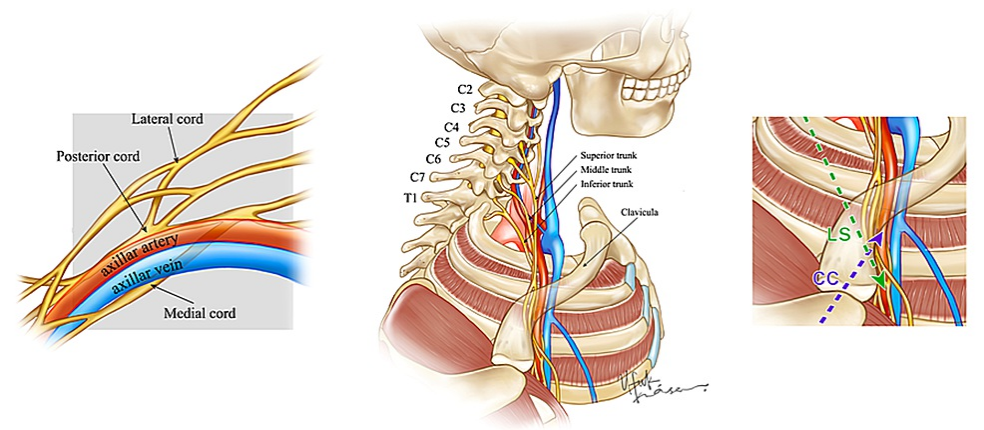
Schematic illustration of brachial plexus anatomy and needle trajectory LS: lateral sagittal; CC: costoclavicular

**Figure 3:**
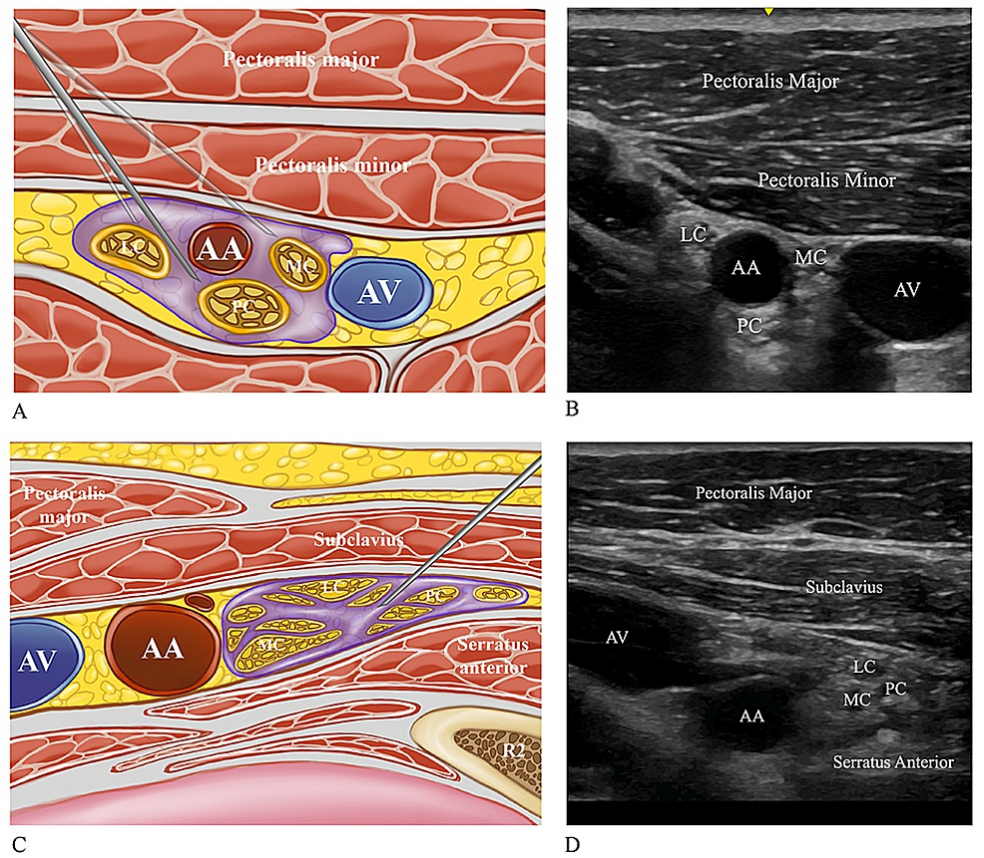
Schematic illustrations related to the injection of local anesthetic for LS-ICB and CC-ICB (A, C), and sonoanatomy relevant for LS-ICB and CC-ICB (B, D) (A) Schematic illustration of where to inject local anesthetic when using an ultrasound-guided LS-ICB. In LS-ICB, the injection is applied around the posterior, lateral, and medial cord. (B) The sonoanatomy relevant for the ultrasound-guided LS-ICB. (C) Schematic illustration of where to inject local anesthetic when using an ultrasound-guided CC-ICB. In CC-ICB, the injection is applied between the posterior, lateral, and medial cord. (D) The sonoanatomy relevant for the ultrasound-guided CC-ICB LS-ICB: lateral sagittal infraclavicular block; CC-ICB: costoclavicular infraclavicular block; PC: posterior cord; LC: lateral cord; MC: medial cord; AA: axillary artery; AV: axillary vein

The patients' ipsilateral arm was abducted for the scan. The head was turned slightly to the contralateral side for the ICB. The US probe was positioned medial to the coracoid process in the sagittal plane in the infraclavicular region, and three cords of the brachial plexus were then visualized. After negative aspiration for blood to exclude any inadvertent intravascular needle placement, and with the needle tip in its target location, an in-plane technique was used to administer bupivacaine (0.5%) around the posterior cord (7 mL), lateral cord (7 mL), and medial cord (6 mL) (Figures 2, 3).

Costoclavicular approach (Group CC)

The US probe was positioned parallel to the clavicle in the midclavicular area, tilted toward the cephalad and the axillary artery, and the three cords were visualized. If the cephalic vein or thoracoacromial artery was visualized, the US probe was slightly tilted toward the cephalad. After negative aspiration for blood, an in-plane technique was used to advance the needle from a lateral to the medial direction, and 20 mL of bupivacaine (0.5%) was administered to the center of the three cords (Figures 2, 3).

Outcome measures after ICB

After the completion of the block procedure (the time of the withdrawal of the needle from the skin was regarded as zero minute), the sensory and motor blocks were evaluated at five, 10, 15, 20, 30, and 45 minutes. The sensory block was evaluated by the pinprick test in the medial, lateral, and posterior cord dermatome area (none = 0, present = 1), while the motor block was evaluated by the modified Lovett rating scale (6 = normal muscular force, 5 = slightly reduced muscular force, 4 = pronounced reduction in muscular force, 3 = slightly impaired mobility, 2 = pronounced mobility impairment, 1 = almost complete paralysis, 0 = complete paralysis) [9]. The motor block cord myotomes were evaluated as a medial cord (thumb adduction = ulnar nerve), lateral cord (elbow flexion = mucocutaneous nerve), and posterior cord (wrist extension = radial nerve). When the score of all dermatomes was 0 on a sensory examination administered 45 minutes after the injection, the block was considered successful. If the block was not successful, a rescue block was planned. The patients still experiencing pain were excluded from the study and converted to general anesthesia. In cases where the pain score was ≥4/10 on the numerical rating scale, dexketoprofen 50 mg was administered as rescue analgesia (a maximum of four times) during the first 24 hours of follow-up.

Primary Outcome Measures

In our study, the block performance time and the motor and sensory block onset time were determined as the primary outcomes. The block performance time was defined as the time from the insertion of the needle into the skin (after obtaining an optimal view on the US) until the block needle was removed from the skin after the completion of the procedure. The motor block onset time was defined as the time when a Lovett score of 5 was achieved in at least one of the three cords in the extremity of the patient undergoing the operation following LA injection. The sensory block onset time was defined as the time from the local injection until no response to the pinprick test in at least one of the three cords in the extremity to be operated on.

Secondary Outcome Measures

Our secondary outcomes were determined as motor and sensory block duration, patient and surgeon satisfaction levels, number of needle redirections, number of needle attempts, number of patients who required a rescue block, and first analgesic requirement. The duration of the motor block was defined as the time interval when the Lovett score was 2 in any of the three dermatomes of the extremity of the patient to be operated on. The duration of the sensory blockade was defined as the time interval when the patient described pain, or when a positive response to the pinprick test was detected in the extremity to be operated on. Patient and surgeon satisfaction was evaluated at the end of the operation day using a visual rating scale (VRS: 0-100; 0 = excessive discomfort, 100 = no discomfort at all). The number of needle redirections was defined as the number of attempts required to withdraw and redirect the needle without complete withdrawal from the skin. The number of needle attempts was defined as the number of withdrawals and redirections of the needle with complete withdrawal from the skin. The number of patients with complete sensory (sensory score = 0), motor (Lovett score = 0), or sensory-motor (sensory score = 0, Lovett score = 0) blocks of all three cords at all time points during the study was also calculated. Complications associated with the block (vascular puncture, hematoma, convulsion, pneumothorax, phrenic nerve paralysis, systemic toxicity, Horner's syndrome, and laryngeal nerve paralysis) were recorded. One week after the surgery, an anesthesia resident called the patients to inquire about complications, such as persistent numbness/paresthesia or motor deficit.

Sample size and statistical analysis

In a previous study involving a similar block, based on the block onset time values (the mean block onset time was 15.4 ± 6 minutes for the retroclavicular approach group and 18.2 ± 5.1 minutes for the coracoid approach group), at least 50 patients were required in each group to attain a confidence level of 95% (1-α), a test power of 80.3% (1-β), and an effect size of 0.503 [10].

The data were analyzed using IBM SPSS Statistics v23 (IBM, Armonk, NY). Normality was checked by the Kolmogorov-Smirnov test. The chi-square test and Fisher's exact test were used to compare categorical variables by groups. The Mann-Whitney U test was used to compare non-normally distributed data by paired groups, and the independent two-sample t-test was used to compare normally distributed data. Friedman's test was used for non-normally distributed data in the comparison of scores according to three or more times. The analysis results were presented as mean ± standard deviation (SD) and median (interquartile range) for quantitative data, and as frequencies for categorical data. The level of statistical significance was set at a p-value of <0.05.

## Results

Of the 100 patients included in the study, 50 underwent CC-ICB, and 50 underwent LS-ICB. The demographic data and the number of patients who required a rescue block were similar between the groups (Table 1). No block-related or surgery-related complications were encountered in either group during the operation and at the one-week follow-up.

**Table 1:** Patient characteristics ASA: American Society of Anesthesiologists; BMI: body mass index; SD: standard deviation; LS: lateral sagittal; CC: costoclavicular

Variables	Group LS (n = 50)	Group CC (n = 50)
Age, years, mean ± SD	41.3 ± 13.93	40.36 ± 13.45
Sex, M/F, n	41/9	42/8
BMI, kg/m^2^, mean ± SD	26.55 ± 3.48	25.47 ± 3.27
ASA class, 1/2, n	29/21	38/12

**Figure 4:**
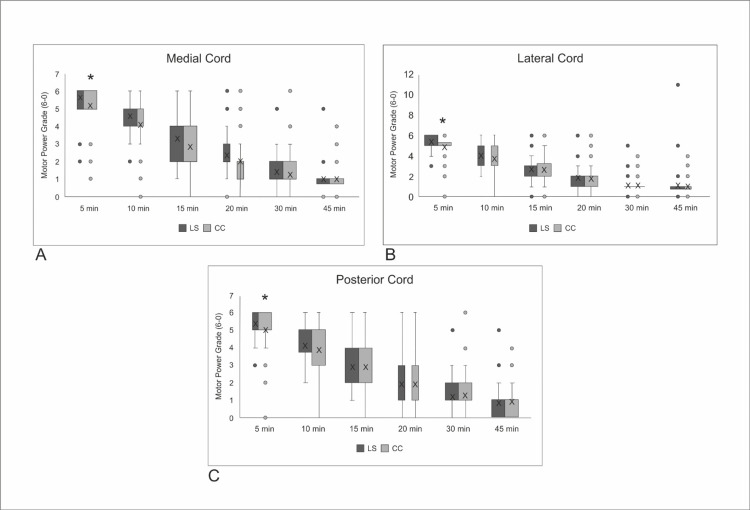
Motor blockade scores according to time in the distributions of (A) the medial cord, (B) the lateral cord, and (C) the posterior cords Data are presented as median (interquartile range) *Statistically significant difference between the groups LS: lateral sagittal; CC: costoclavicular

The block performance time and the sensory block onset time were shorter and the number of needle redirections was lower with the CC approach (p: <0.05) (Table 2). Comparison of the sensory and motor block scores of both techniques by the cords revealed a significantly lower five-minute motor block score for the medial (p = 0.003), lateral (p = 0.003), and posterior (p = 0.049) cords in the CC approach. No difference was noted between the groups in terms of the motor block score at the subsequent time intervals (Figure 4). The sensory block scores were similar for the two techniques for the posterior cord at all time intervals. For the medial and lateral cords, the five-minute (p = 0.009, p = 0.046, respectively) and the 10-minute sensory block scores (p = 0.030, p = 0.033, respectively) were significantly lower with the CC approach.

**Table 2:** Block performance data The block performance time and the sensory block onset time were shorter and the number of needle redirections was lower in group CC *Statistically significant difference between groups CI: confidence interval; LS: lateral sagittal; CC: costoclavicular

Variables	Group LS (n = 50)	Group CC (n = 50)	P-value
Block performance time, minutes, median (interquartile range) [95% CI]	3 (2.5–3.37) [3–3.2]	2 (1.52–2.3) [2–2.2]	<0.001*
Number of needle redirections, median (interquartile range)	3 (2.75–4)	2 (1–2)	<0.001*
Number of needle attempts, median (interquartile range)	1 (1–1)	1 (1–1)	1.000
Motor block onset time, minutes, median (interquartile range) [95% CI]	5.4 (5–6.1) [5–6]	5 (4–6.48) [5–7]	0.092
Sensory block onset time, minutes, median (interquartile range) [95% CI]	5 (4.49–6) [5–6]	4 (3.88–6) [4–5]	0.022*
Motor block duration, minutes, median (interquartile range)	315 (287.5–427.5)	300 (250–400)	0.208
Sensory block duration, minutes, median (interquartile range)	345 (300–462.5)	300 (295–400)	0.232
First analgesia requirement time, minutes, median (interquartile range)	400 (320–500)	400 (300–480)	0.266
Patient satisfaction, median (interquartile range)	100 (90–100)	90 (90–100)	0.163
Surgeon satisfaction, median (interquartile range)	100 (90–100)	90 (90–100)	0.390
Rescue block, n (%)	4 (8)	4 (8)	1.000

Comparison of the number of patients with a complete motor block revealed no difference between the two techniques at any time interval, whereas the number of patients with a complete sensory block was significantly higher in the CC approach at five and 10 minutes (p = 0.027, p:<0.001, respectively). The number of patients with complete motor and the sensory block was similar for the two techniques at all time intervals.

## Discussion

Our study findings indicated a shorter block performance time and a lower number of needle redirections for the CC approach compared to the LS approach. The sensory block also started faster with the CC approach. The number of patients who achieved a complete sensory block was higher in the first 10 minutes with the CC approach. The motor block onset time and the motor and sensory block times were similar in both groups. No differences were detected in terms of the first analgesic requirement time, the patient and surgeon satisfaction levels, and complications.

The use of single/multiple-injection techniques for CC and LS approaches has been described in the literature [2,6,11]. In our study, we used a single-point injection technique for the CC approach and a triple injection technique for the LS approach. The results showed that the number of needle redirections was higher and the block performance time longer for the LS approach. A successful block could be achieved in a shorter time with a single injection in the CC approach due to the compact structure of the plexus. The requirement for multiple injections due to the scattered location of the cords in the LS approach may be the reason for the differences observed between these two groups. The deeper location of the cords and the inability to visualize all of them at the same time in the LS approach may have contributed to the results that favored the CC approach [7,12]. In fact, block performance time was statistically significant but clinically unimportant in routine practice.

Leurcharusmee et al. used lidocaine + bupivacaine (35 ml) and showed that the CC approach and the LS approach had similar sensory and motor block onset times [8]. In our study, the sensory block onset time was shorter with the CC approach compared to the LS approach. This shorter duration may be attributed to the fact that the sheath of the brachial plexus is denser anatomically and surrounds the cords more tightly in the proximal area and more loosely in the distal area [13,14]. Songthamwat et al. obtained results similar to ours using ropivacaine (25 ml), and they explained their findings as reflecting a decrease in extraneural diffusion and an increase in intraneural diffusion as a result of the administration of LA under the dense sheath [7]. The similar block onset times found in the study by Leurcharusmee et al. may be related to the use of different volume and LA mixtures. When the cords were analyzed separately, the sensory and motor block (except for the posterior cord) was initiated more rapidly with the CC approach. Although Leurcharusmee et al. used a larger volume of LA mixture, they obtained results similar to ours [8].

We conclude from our results that the CC approach should be favored, even at a low anesthetic volume, with the compact location of the dense plexus sheath and cords. A close examination of the CC anatomy has shown a high rate (94%) of intracompartmental septum presence in this region [15]. Layera et al. showed a faster onset of the block with a double injection-CC approach compared to a single-injection one [16]. In our study, the sensory block onset time was shorter despite a single injection. Since the available literature lacks sufficient data, more studies comparing the double-injection CC approach and the LS approach are required.

The current literature shows that a lower LA volume (20 mL) is used for the CC-ICB compared to the LS-ICB approach (20-35 mL) [6,8,17,18]. High doses of LA can lead to LA-related systemic toxicity and postoperative patient discomfort due to excessive paralysis [19]. Moreover, this is also irritating for surgeons since paralysis delays the assessment of nerve function after surgery, especially after soft-tissue surgery that can damage peripheral nerve function [20]. Although we expected high satisfaction scores in the CC approach, we found no differences between the groups. This may reflect the fact that each patient had only one approach and therefore could not compare the two approaches, and the small number of patients (40%) who achieved a complete motor block in both blocks, which may be related to our preference for low volumes and the type of LA used. In addition, the administration of sedation to patients during the block and surgery may also have affected the satisfaction scores.

Various different approaches (such as coracoid, LS, and vertical approaches) have been described for ICB [12,21,22]. Although studies have been conducted on the block dynamics, dose volume, and complications of the LS approach and the findings have been introduced into routine practice, the CC approach dynamics have not been adequately investigated. In this study, we demonstrated that the cords could be blocked more rapidly with the CC approach and that the block was administered faster and required a fewer number of needle redirections. There was no increased incidence of pneumothorax with the CC approach. This may be feared as a complication by some clinicians because the needle is directed towards the underlying lung with this approach. The lack of complications, despite manipulations close to anatomically important structures, may encourage clinicians to use this block more often in daily practice. However, further controlled studies with large sample sizes are still required in the future to provide a more comprehensive data analysis for comparison of the complications. The limitations of our study include a failure to determine the "surgery readiness" criteria, rather than waiting 45 minutes to allow the surgery to begin, and the evaluation of patient satisfaction at the end of the day, rather than after the administration of the block.

## Conclusions

Our findings showed that the CC approach provided a shorter performance time and a faster onset of the sensory block compared to the LS approach, while no complications developed in either technique, and each resulted in similar patient and surgeon satisfaction levels. Further studies of the two approaches (CC and LS) involving different dosages and injection techniques are warranted to assess outcomes in terms of reliability, satisfaction, and efficiency.
